# Empowering beyond Pain: Pain Neuroscience Education Interventions in Breast Cancer Survivorship Care

**DOI:** 10.3390/cancers16162806

**Published:** 2024-08-09

**Authors:** Marco Balordi, Paola Tiberio, Matteo Castaldo, Alessandro Viganò, Flavia Jacobs, Alberto Zambelli, Armando Santoro, Rita De Sanctis

**Affiliations:** 1 Department of Biomedical Sciences, Humanitas University, 20072 Pieve Emanuele, Italy; marco.balordi@st.hunimed.eu (M.B.); paola.tiberio@cancercenter.humanitas.it (P.T.); matteo.castaldo@poliambulatoriofisiocenter.com (M.C.); flavia.jacobs@cancercenter.humanitas.it (F.J.); alberto.zambelli@hunimed.eu (A.Z.); armando.santoro@cancercenter.humanitas.it (A.S.); rita.de_sanctis@hunimed.eu (R.D.S.); 2 Medical Oncology and Hematology Unit, IRCCS Humanitas Research Hospital, 20089 Rozzano, Italy; 3 Department of Health Science and Technology, Center for Pain and Neuroplasticity (CNAP), Sensory Motor Interaction (SMI), Faculty of Medicine, Aalborg University, 9220 Aalborg, Denmark; 4 Clinical Psychology, Clinical Psychophysiology and Clinical Neuropsychology Labs, Parma University, 43126 Parma, Italy; 5 IRCCS Fondazione Don Carlo Gnocchi, 20148 Milan, Italy

**Keywords:** pain neuroscience education, breast cancer, pain, biopsychosocial model, biomedical education, neuropathy

## Abstract

**Simple Summary:**

Chronic cancer-related pain, caused by surgery, radiotherapy, or systemic treatments, affects almost half of all breast cancer patients, and current therapeutic options are insufficient. Pain neuroscience education (PNE) has provided relief in many chronic pain syndromes but has been rarely applied in the cancer field. PNE is also without side effects and could therefore be implemented at different moments of the patient’s journey. We analyzed trials investigating PNE efficacy for managing breast cancer-related pain, identifying methodological issues that should be addressed in future studies to obtain high-quality data.

**Abstract:**

Chronic pain is a common consequence of breast cancer (BC) and its treatments. Pain neuroscience education (PNE) is a non-pharmacological intervention that adopts a biopsychosocial approach and has already been proven to be effective for different chronic pain syndromes. The present review aims to critically assess clinical trials comparing the efficacy of PNE to traditional biomedical education (BME) in reducing BC-related pain and improving quality of life. We conducted a literature search in scientific databases, including all studies regarding PNE use specifically for BC-related pain. Ongoing randomized controlled and observational studies were identified from ClinicalTrials.gov and congress proceedings. A total of eight clinical trials met the review criteria. The participants were all administered physical therapy and assigned to receive either BME or PNE interventions. Among the completed clinical studies, one reported no statistically relevant differences between the two groups, whereas the other showed lower levels of pain-related indexes in the PNE population compared to the BME one. While the current literature is inconclusive regarding the effectiveness of PNE for managing BC pain, we strongly support the need for further trials, as PNE could empower BC patients in both prevention of and coping with pain, offering the advantage of having no side effects.

## 1. Introduction

The global population of breast cancer survivors (BCSs) is progressively growing due to advancements in early diagnosis and reduced mortality [1]. This expands the evolving field of survivorship care for breast cancer patients who have completed primary treatment, aiming not only to prolong lifespan but also to enhance quality of life (QoL) across various domains [2]. In this context, persisting pain emerges as a significant detrimental element, with approximately 20% of BCSs reporting moderate-to-severe pain [3]. Chronic cancer-related pain can stem directly from the disease or be secondary to treatments, such as post-mastectomy pain, chemotherapy-induced neuropathy, aromatase inhibitor-induced musculoskeletal pain, and radiotherapy-induced pain [4]. Regardless of its origin, chronic pain can be considered as a complex condition influenced by psychological and social factors, aligning with the biopsychosocial model [5]. The relationship between chronic cancer pain and psychological and biological factors is intricate.

Indeed, Zaza and Baine [6] reported that chronic cancer pain was strongly associated with psychological distress and moderately with poor social support, while they found inconclusive evidence for the coping mechanisms. On the other hand, further studies [7] showed stronger correlations with coping-related cognitive domains. In particular, a cross-sectional study on BCSs found that patients with higher scores in the domains of perceived injustice (PI) [8] and pain catastrophizing (PC) [9] were more likely to experience long-lasting higher pain levels. Furthermore, both PI and PC influenced the levels of fatigue and sleep disturbance [7]. This could trigger a noxious mechanism in which sleep deprivation, pain, and psychological distress reinforce each other. This model fits the case of breast cancer, as patients often have to cope with a new body image [10] and readjustments in their roles within the family [11]. Nonetheless, the current approach to managing chronic cancer pain primarily relies on pharmacological treatments, but there is growing interest in alternative or complementary educational interventions that have proved their effectiveness in improving cancer-related pain management [12]. The aim of such educational programs is to provide patients with the necessary tools to understand and cope with pain, with the advantage of the low risk associated with non-pharmacological interventions. Pain neuroscience education (PNE) is an educational intervention centered on the biopsychosocial model, which uses a multimodal approach to address patient’s beliefs about pain and improve coping strategies [10]. It comprises theoretical interactive lessons about the physiology of chronic pain, with a focus on how the behavior and attitude of the patient can influence daily pain levels. This represents a broadening of perspective compared to previous interventions based on pharmacological/practice treatments, which typically view pain solely as a sign of tissue or nerve damage to be managed with analgesics [10] and exercise [13]. A biomedical approach may inadvertently alarm cancer patients with new chronic pain, potentially perceived as a recurrence rather than a sensitization. On the other hand, PNE may reduce the tendency to ruminate about painful sensations [10] (e.g., if I experience pain, something must have gone wrong with my disease) by contextualizing pain as a multifactorial phenomenon, thus empowering patients to actively contribute to their healing journey.

The body of literature regarding PNE is well established regarding benign musculoskeletal pain [14] but still in progress in the BCS field [4]. The aim of this review is to revise the state of the art of PNE in reducing chronic pain in breast cancer patients by analyzing and comparing results from all clinical studies and protocols published to date.

## 2. Materials and Methods

A narrative approach was chosen for the present review, considering the limited number of trials to date regarding the application of PNE for reducing pain in BCSs.

Articles were sourced from PubMed using specific keywords related to “breast cancer” OR “breast cancer survivor” OR “breast cancer surgery” OR “mastectomy” AND “pain neuroscience education” OR “PNE” OR “chronic pain” OR “biopsychosocial model” OR “educational intervention”.

Only trials focusing on PNE’s effectiveness for breast cancer-related pain were included, while other studies on pain from other causes (e.g., musculoskeletal disorders) were excluded. A total of 8 articles were selected for the analysis, covering 3 different clinical trials (Educan [13], Manfuku et al. [15], and PaiNEd [10]) conducted between 2019 and 2023.

In addition, yet-to-be-published studies from ClinicalTrials.gov [16,17,18,19,20] as well as congress proceedings from international cancer conferences that took place in the last two years are discussed in a separate section. All the reviewed publications were written in the English language.

## 3. PNE Interventions in BCS Patients

The use of PNE for breast cancer-related pain management constitutes a relatively new field; to date, only a few publications have investigated its effectiveness in reducing chronic pain, namely, the EduCan trial by De Groef et al. [13], the PaiNEd trial by Fernández-Gualda et al. [10], and the study by Manfuku and colleagues [15].

### 3.1. Study Designs

A double-blinded randomized control design was the study design of choice for both the EduCan and PaiNEd trials [10,13], while Manfuku et al. conducted a retrospective case–control study [15].

All three groups compared the outcomes of PNE with biomedical education (BME)—another educational intervention that explains the experience of pain from a tissue-based and biomechanical point of view [13]. In addition, all patients enrolled in the three studies received physiotherapy sessions that implemented exercises and manual techniques [10,13,15]. Patients were divided into two arms based on the PNE or BME intervention received [13,15]; however, it is notable that the ongoing PaiNEd trial features a third arm including a control group of breast cancer patients who do not receive any physical therapy or educational intervention, except for an informational leaflet [10].

Randomization provides an advantage in preventing bias by balancing patients’ characteristics so that differences in outcome can be attributed to the intervention [21], thus providing an excellent tool for supporting cause–effect relationships. Also, the blinding of participants and research teams is instrumental in providing good-quality data. However, as Dams et al. stated, we should take into consideration that in the EduCan trial the same physiotherapist delivered both PNE and BME and that this person might be biased about the superiority of one intervention over the other [22].

By contrast, Manfuku et al. conducted a retrospective case–control study, which lacks the advantage given by randomization [15]. This may have caused differences associated with the post-operative treatment received by the patients. Nevertheless, the BME and PME arms were analyzed and showed no statistically significant differences in demographic data, type of surgery, or post-operative treatments received [12]. The second limitation of the study by Manfuku and colleagues is a mismatch in temporality between the two groups, as the BME arm received the intervention between April 2016 and August 2017, while the PNE population received it between October 2017 and February 2019 [15]. The authors cannot exclude the possibility that this may have led to some changes [23] in terms of patient expectations [15], the clinical setting of the hospital, or the spontaneous evolution of symptomatology over time.

### 3.2. Strategies of Intervention

The procedures used to administer the interventions differed among the trials. In the EduCan trial, BCSs first underwent a 4-month intensive phase in which they received physical therapy once or twice a week along with three PNE sessions [13]. This period was followed by a maintenance phase in which they received three sessions of both physical therapy and PNE at 6, 8, and 12 months after surgery, each session lasting 30 min and conducted one-on-one by a physiotherapist [13].

Similarly, the breast cancer patients enrolled by Manfuku et al. underwent a physical therapy session for 3 months every one or two weeks, each accompanied by a PNE session [15], both administered one-on-one in person and lasting 40 min. However, unlike the EduCan trial, no reinforcement sessions for maintenance were provided post-surgery.

Finally, in the ongoing PaiNEd trial, patients in the first and second intervention arms underwent 60 min of supervised exercise and one manual therapy session of 30 min every 2 weeks for 8 weeks, with the third arm not receiving any physical therapy [10]. Educational interventions varied significantly, with PNE delivered via a mobile application (the PaiNEd app), comprising seven lessons of 20 min each, while BME was provided with an informational leaflet to the patients belonging both to the second arm, the one also receiving physical therapy, and to the third arm, the one not receiving physical therapy [10].

Despite the potential superiority of one-on-one, in-person interaction, the efficacy of a mobile application should not be discarded a priori, given the high patient compliance, acceptability, and overall satisfaction observed in previous studies [24].

**Figure 1:**
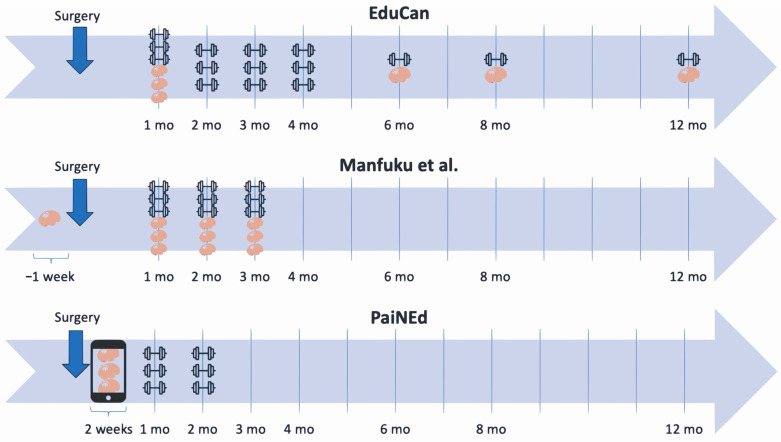
Timeline visually representing the time of educational interventions (brain) and physical therapy (dumbbell) [15].

In addition, the timing of PNE administration differed across the trials, starting right after surgery in the EduCan trial [13] and one week before surgery in the trial by Manfuku et al. [15]. In contrast, the PaiNEd trial provided the PNE sessions at least 6 months after surgery and/or adjuvant therapy, with potential delays of up to two years post-adjuvant therapy [10]. The authors suggest that patients in the early perioperative period could be less receptive to PNE due to heightened stress and poor cognitive resources, which are common in this phase [10]. The difference in the timing of the interventions among the different trials is summarized in Table 1 and conveyed visually in Figure 1.

Regarding the content of the interventions, physical therapy ranged from rehabilitation exercises to manual techniques, with the aim of improving mobility and strength. Specifically, the EduCan trial included specific exercises for the upper limbs and manual techniques to restore range of motion and function, in addition to general recommendations to moderately increase the level of physical activity at home [13]. Similarly, patients in the trial by Manfuku et al. performed stretching and strength exercises, along with scar tissue massage, and received a leaflet encouraging them to continue shoulder-specific exercises at home [15]. Both trials also provided guidance on lymphedema prevention [13,15]. Lastly, the PaiNEd trial features a physiotherapy program including resistance and aerobic exercises, as well as manual therapy [10].

Even more importantly, the content of the PNE sessions in all the trials was homogeneous, addressing the distinction between acute and chronic pain and explaining how pain could be considered a product of the brain [10,13,15], that is to say, a danger signal alerting the system of a potential threat, which may persist even after the resolution of the initial damage, as in the case of chronic pain [4].

The association of physical therapy (both manual and exercise interventions) with PNE may change beliefs regarding and the meaning of the physical therapy interventions, helping patients to change wrong beliefs and attitudes towards pain during exercise or during daily activities.

These interventions underscore the significance of lifestyle and emotional factors in shaping pain experience, emphasizing the importance of coping skills and providing information on pain-related side effects of breast cancer treatment [10,13,15]. Conversely, patients who received BME either in a leaflet or an in-person format were given explanations about how treatment and surgery could cause tissue damage and subsequent pain [10,13,15].

Overall, the strategies employed in these trials could be useful for addressing the potential efficacy of PNE for reducing chronic pain in BCSs. While double-blinded randomized control trials are considered the gold standard [21], caution is warranted regarding blinding effectiveness due to the nature of the studies [22]. Moreover, future data from the PaiNEd trial will suggest whether providing PNE is more beneficial in the perioperative phase or later.

### 3.3. Assessment Methods

The changes in outcomes evaluated at the end of the educational interventions were all related to the different domains of pain and function. The only primary outcome for the EduCan trial was pain-related disability [13], measured with a Dutch version of the pain disability index [25,26]. Similarly, the sole primary outcome for the PaiNEd trial is perceived pain, assessed with a visual analogue scale (VAS), that is, a 0–100 mm line with 0 representing no pain and 100 representing the worst pain imaginable (or 0–10, depending on the fraction) [10]. Finally, the primary outcomes for Manfuku et al. were pain intensity and interference, measured with the brief pain inventory (BPI) [15], as well as central sensitization-related symptoms and pain catastrophizing, assessed with the central sensitization inventory (CSI) and the pain catastrophizing scale (PCS), respectively [15].

A common limitation across all three trials was the reliance on self-assessment for primary outcomes, which may limit the empirical value of the subjective data collected [10,13,15]. Furthermore, any difference among the implemented indexes makes it harder to draw comparisons between the studies.

Secondary outcomes, on the other hand, included both self-assessment questionnaires and parameters that could be evaluated by physicians or through specific tests [10,13,15]. De Groef et al. used the CSI questionnaire implemented with quantitative sensory testing to evaluate the response to different sensory stimuli [27], such as a change in perception of pressure or temperature stimuli. By contrast, Manfuku et al. measured the range of motion (ROM) and handgrip to evaluate function on the operated side and evaluated the presence of lymphedema by measuring the circumference of the operated arm at critical points [15]. Finally, the secondary outcomes in the PaiNEd trial can be considered the most comprehensive. The study includes subjective answers from questionnaires regarding PCS, CSI, kinesiophobia (Tampa Scale for Kinesiophobia), and QoL (EORTC QLQ C30) [10]. Indeed, PaiNEd also features more objectives by collecting measurable data, including active range of motion, function via the 6 min walking test, handgrip strength, and deep neck flexor endurance. In addition, body composition and inflammatory status are also assessed via bioelectrical impedance analysis and salivary cortisol and interleukin 6 (IL-6), respectively [10]. An overview of the different scales used for measuring primary and secondary outcomes is provided in Table 2.

Beyond the differences in primary and secondary study outcomes among the trials, all the statistical analyses in the three trials were performed in a robust manner. In the randomized studies PaiNEd and EduCan [10,13], statistical analyses were carried out as intention to treat. The mean change from baseline to post-intervention timepoints (including follow-up timepoints) was assessed for each primary and secondary outcome. Descriptive statistics in all the trials employed means and standard deviations, median and range values, or frequencies and percentages. 

### 3.4. Populations Analyzed

The populations analyzed in all the trials shared common characteristics, including sex, a diagnosis of early breast cancer, and either scheduled or completed surgery and/or adjuvant therapy. The PaiNEd trial specifically excludes patients who have not completed adjuvant treatment for at least 6 months [10]. The studies included patients older than 18 [10,13] or 20 years [15] and younger than 75 [10] or 79 [15] years old. An additional inclusion criterion for the PaiNEd trial is having pain in regions related to the tumor area, with VAS ≥ 4 (range: 0–10) [10]. This ensures the inclusion of patients experiencing clinically significant chronic pain at least 6 months post-surgery and/or adjuvant therapy.

Regarding cancer stage, all trials excluded metastatic disease (stage IV) [13,15]. Furthermore, the study run by Manfuku et al. took into account the etiology of pain, limiting inclusion to those with persistent post-mastectomy pain and excluding other pain conditions related to treatment, such as chemotherapy-induced peripheral neuropathy or arthralgia due to aromatase inhibitors [15]. This last selection criterion could provide a more homogeneous population in terms of pain type and its causes. With a similar rationale but considering chronic pain conditions prior to surgery, Fernández-Gualda et al. excluded from their study all participants who had chronic pain in the head and neck and in brachial and shoulder areas before surgery [10]. Taking into account all the abovementioned inclusion criteria, the final numbers of eligible patients included in the three trials were 72 for PaiNEd [10], 184 for EduCan [13], and 118 for the study of Manfuku et al. [15].

### 3.5. PNE Efficacy: Main Results

The completed trials showed different results on the efficacy of PNE in reducing cancer-related chronic pain in breast cancer patients. First of all, patients in the EduCan trial did not show any relevant difference between the control and intervention groups in pain-related disability at 12 months after surgery, nor for any of the secondary outcomes. The lack of statistically significant change remained consistent at 4, 6, 8, and 18 months post-operatively [22]. Further secondary analyses of the EduCan trial by De Groef. et al. showed no differences between PNE and BME in other work-related outcomes, such as median time before returning to work and estimation of own ability to work [26].

Several hypotheses were formulated to explain these findings. More than half of the patients experienced low pain intensity (VAS around 30/100) at the time of PNE administration, which could have hindered the relevance of the intervention, preventing a paradigm shift [27]. In other words, this trial may have involved patients without a relevant chronic pain condition, masking the non-efficacy results found in this sample. This aspect will be addressed in the PaiNEd trial by the addition of an inclusion criterion of VAS ≥ 4 in the operated area [10]. Furthermore, in the EduCan trial, the effects of adding PNE could have been masked by the ceiling effect, as both groups could have already benefited from physical therapy and educational intervention (either PNE or BME), possibly making it harder to register any further improvement in outcomes [10]. This may be supported also by the fact that attending rigorous physiotherapy may have diverted the attention of patients from the immediate benefits of physical therapy, shifting their focus from the reconceptualization required by PNE [22].

On the other hand, Manfuku et al. reported a higher efficacy for PNE compared to BME in improving both primary and secondary outcomes 1 year after surgery [15]. In particular, PNE was found to significantly decrease (*p* < 0.05) scores for BPI, CSI, and PCS compared to BME [15,27]. The effect sizes were moderate (r = 0.31) for BPI intensity and small for the other scores (0.20 ≤ r ≤ 0.29) [15].

The reasons behind the inconsistencies between these promising results and those obtained with the EduCan trial remain to be further addressed. The main difference between the selected population in the Manfuku et al. study and those in the other studies is the exclusion of patients reporting treatment-related pain other than persistent post-mastectomy pain, thus providing a more homogenous population in terms of the underlying nociceptive mechanism [15,22].

### 3.6. Study Limitations

When addressing the feasibility of PNE, it is crucial to recognize that reconceptualizing pain in an oncologic population could be harder compared to other patients affected by chronic pain syndromes, as cancer survivors tend to associate pain with increased fear of recurrence [12].

Additionally, these studies may have inherent limitations related to the nature of the interventions. First of all, a sampling bias may have occurred, since willingness to participate in the study may have unknowingly selected patients with certain traits, such as openness to change or belonging to certain age cohorts [15], thus compromising the representational validity of the population [26]. Secondly, educational interventions such as PNE might fall into the category of “complex” interventions [26]. These interventions are characterized by challenges in identifying the active ingredients and ensuring consistent administration across different settings [38]. While randomized control trials, such as PaiNEd and EduCan [10,13], might represent the best option to support causality [21], in the case of complex interventions such as PNE, some adjustments may be needed to preserve their validity. This might involve monitoring the different responses of the various subgroups or adapting the content of the intervention to each subpopulation.

Nonetheless, when comparing the different study designs and interventions, the PaiNed trial [10] emerged as the most promising for the reliable assessment of the effectiveness of PNE in the management of breast cancer-related pain for many reasons. First of all, the PaiNEd trial added as an inclusion criterion a threshold for the presence of pain (VAS ≥ 4). Furthermore, the authors explored the effect of PNE as a standalone intervention (thus avoiding the possible ceiling effect of physiotherapy), evaluated a most comprehensive panel of secondary outcomes (incorporating subjective answers from questionnaires, measurable physical data, and inflammatory status), and included a follow-up assessment after the end of the intervention. However, we must highlight the smaller sample size (*n* = 72, 24 patients per arm) of the PaiNEd trial compared to the EduCan (*n* = 184) [13] and Manfuku ones (*n* = 118) [15].

### 3.7. Unpublished Studies

To date, five yet-unpublished studies investigating the use of PNE for the management of breast cancer pain can be found in the online register for clinical trials [18]. These studies are being conducted by research groups in different countries [16,17,19,20,39], underscoring the current interest in PNE as one of the available options for alleviating cancer-related chronic pain.

The study designs are either randomized controlled trials, with or without blinding, or prospective observational studies [16,17,19,20,39]. Regarding recruitment, in all the studies, the inclusion criteria select only patients who have completed primary treatment, such as radiotherapy and/or chemotherapy, at least 3 months prior to starting PNE intervention [16,17,19,20,39]. This could limit the onset of confounding side effects from previous treatments [40] during PNE intervention while also allowing a higher rate of participation thanks to the improved wellbeing of patients. Obviously, the presence of pain is a primary criterion for enrolment, with all the studies apart from one [19] setting a threshold for recruitment, most of them using the VAS (VAS > 40/100 [16] VAS > 3/10 [20], or VAS > 30/100 [39]) or the BPI (BPI > 3/10 [17]) scales. Demographic criteria include patients above 18 [16,17,19,20] or 25 [39] years old, with two studies setting an upper limit of 65 [19,39]. In the latter case, the age limit is most likely set to enhance receptivity to the intervention, which is often provided online, as well as to decrease the likelihood of comorbidities unrelated to breast cancer. On the other hand, BCSs with metastatic disease are excluded from all the trials [16,17,19,20,39]. The exclusion criteria generally exclude patients with metastatic disease, breast cancer-unrelated pain syndromes [16,17,39], or medical diagnoses affecting other systems [19].

The intervention arms receive varying numbers of PNE sessions, ranging from a minimum of three to a maximum of six [16,17,19,20,39]. Arguably, the difference in the number of sessions might be useful to define a minimum effective dose for PNE. Along with that, the extent of PNE efficacy might be inferred by comparing the results of trials pairing PNE with physical therapy [16,19,20,39] to those opting for PNE only [17]. Whenever present, the control arms receive either no intervention [19], a leaflet with biomedical information [20], or actual sessions of BME [16,17]. A comparison of the different study designs is presented in Table 3.

Primary outcomes are related to pain (e.g., VAS, BPI, and numerical rating scales [NRSs]) in all the trials [16,17,19,20,39], with the exception of one also evaluating QoL (Functional Assessment of Cancer Therapy–Breast + 4 [FACT-B + 4] [41], EORTC QLQ C30 [19]).

Secondary outcomes encompass a comprehensive range of factors, including sensitivity thresholds, psychological domains (e.g., depression and anxiety), central sensitization, cognitive appraisals (e.g., pain catastrophizing and perceived injustice), upper limb functionality (e.g., ROM, kinesiophobia, and lymphedema), type of pain (e.g., neuropathic or nociplastic), treatment adherence, and healthcare expense [16,17,19,20,39]. Especially noteworthy, three studies also measure sociodemographic items, including education, occupation, and marital status, in addition to personal characteristics, such as body mass index, tobacco or alcohol use, and comorbidities [17,39].

Lastly, assessment timing starts from the baseline in all studies [16,17,19,20,39], with some providing reassessments during or right after the intervention [16,17,19,39]. The most comprehensive trials include follow-ups at 6, 12, or even 24 months [17,19,20,39].

In addition, among these unpublished trials, some encouraging results by Lahousse et al. [20] were shared at the 14th European Breast Cancer Conference [42]. They compared PNE combined with behavioral graded activity to usual care, reporting a statistically significant decrease not only in BPI, but also in CSI, PCS, and pain vigilance and awareness [43]. Positive results were consistent both at the end of the intervention and after 3 months of follow-up. However, no improvements were reported in patients’ QoL (health-related QoL [44]) nor in endogenous pain modulation.

## 4. Conclusions

The current state of research supporting the potential application of PNE for breast cancer patients is still evolving, emphasizing the need for future well-structured trials. This could be achieved by identifying whether there are subgroups of patients who could benefit more from PNE [27], either due to sociodemographic factors (e.g., age and education status) or due to the type of pain experienced (e.g., persistent post-mastectomy pain) [15]. For the same reason, trials targeting younger segments of the breast cancer patient population [15], such as those with triple-negative breast cancer, could be interesting, as young and active patients could be more receptive to educational interventions promoting their wellbeing and active role in the world. Following this rationale, our group is currently conducting a feasibility study on the administration of PNE to early triple-negative breast cancer patients with pain syndromes [45]. The aim will be to establish whether PNE is helpful in diminishing pre-existing pain or in preventing the unfolding of chronic pain and its sequelae in this specific population.

Methodologically, double-blinded randomized controlled designs should be considered optimal [21] for future studies. However, randomization could raise ethical issues in the case that the control arm does not receive any intervention at all [46], thus conflicting with the principle that study participants in control groups should be treated with the best available proven methods [46]. Therefore, if a control arm is foreseen, BME or physical therapy-only sessions should be administered.

Furthermore, the implementation of inclusion criteria regarding a threshold for the presence of pain (e.g., NRS > 4 or VAS > 4) could ensure the relevance of the intervention at the time of PNE administration [22,45] as well as allow assessment of whether the intervention causes any statistically significant changes from the baseline [10,45]. Regarding the timing of administration, while there is a possibility that excessive stressors in the perioperative period may affect patients’ cognitive resources [10], it is also true that the tools provided by PNE could improve active coping in a critical period, thus preventing the onset of chronic pain.

Concerning the modality of PNE administration, an adequate number of sessions should be provided to guarantee enough exposure to the concepts of PNE, which challenge patients’ pre-existing beliefs about pain [4,12]. Secondly, since the ceiling effect of physiotherapy could mask any further improvement in pain and function [10], future trials may explore the effect of PNE as a standalone intervention, as in the case of the comparison of the PNE group with the control arm in the PaiNEd trial and in our study [10,45]. Furthermore, especially for young patients under treatment, the timing and methods of PNE administration must be carefully considered. In fact, due to their chemotherapy-busy schedules, patients may prefer a mobile application instead of a one-on-one, in-person interaction. Moreover, in the case of breast cancer patients undergoing neoadjuvant treatment, the timing of surgery should be taken into account, since the surgery-related pain could worsen patients’ painful syndromes, possibly hiding the effects obtained by PNE during the pre-operative therapy. 

Then, as previously stated, assessment of outcomes should be performed by combining self-evaluation questionnaires with some measurable variables (e.g., ROM and levels of inflammatory markers) to obtain more comprehensive data. Furthermore, to check for any potential long-term benefit, it would be advisable to integrate follow-up assessments at 6 months or longer after the end of the intervention [10,13,45].

Considering all the variables mentioned above, the PaiNed trial appears promising in terms of providing reliable results on the possible effectiveness of PNE for breast cancer-related pain management. We also look forward to the results of the five unpublished trials registered on ClinicalTrials.gov. Collectively, the data generated from these studies hold the promise of establishing a solid foundation for evaluating the effectiveness and scope of PNE in addressing breast cancer-related pain. Such findings could potentially pave the way for a change in the management of chronic pain in BCSs, or, in case of negative results, give more space for the exploration of alternative treatment options.

## Figures and Tables

**Table 1:** Table illustrating the different timings of the interventions. Post-op: post-operative; PNE: pain neuroscience education; BME: biomedical education.

Interventions	EduCan	Manfuku et al. [15]	PaiNEd
Physical therapy	**Intensive phase:** Up to 4 months post-op 1–2 sessions per week (30 min)	Up to 3 months post-op 1 session (40 min) every 1–2 weeks	2 months post-op 1 session every 2 weeks (60 min exercise + 30 min manual therapy)
	**Maintenance phase:** 3 sessions at 6, 8, and 12 months post-op (30 min)		
PNE	**Intensive phase:** Up to 4 months post-op 3 sessions (30 min)	**Pre-Operative:** 1 week before 1 session (40 min)	2 weeks post-op 7 lessons on PaiNEd App (20 min)
	**Maintenance phase:** 3 sessions At 6, 8, and 12 months post-op (30 min)	**Post-Operative:** Up to 3 months post-op 1 session (40 min) every 1–2 weeks	
BME	**Intensive phase:** Up to 4 months post-op 3 sessions (30 min)	**Pre-Operative:** 1 week before 1 session (40 min)	Leaflet was handed out post-op
	**Maintenance phase:** 3 sessions At 6, 8, and 12 months post-op (30 min)	**Post-Operative:** up to 3 months post-op 1 session (40 min) every 1–2 weeks	

**Table 2:** Scales used to measure outcomes in the different trials (primary outcomes are highlighted with an asterisk).

EduCan	Manfuku et al. [15]	PaiNEd
Pain disability index * [25]	Brief pain inventory * [28]	Visual analogue scale * [29]
Central sensitization inventory [30]	Central sensitization inventory * [30]	Pain catastrophizing scale [9]
Quantitative sensory testing [31]	Pain catastrophizing scale * [9]	Central sensitization inventory [30]
	Range of motion	Kinesiophobia [32]
	Handgrip	Quality of life [33]
	Lymphedema	Quality of life [34]
		Active range of motion
		6 min walking test [35]
		Deep neck flexor endurance [36]
		Bioelectrical impedance analysis [37]
		Salivary cortisol and IL-6

**Table 3:** Comparison between the different study designs of the unpublished trials from ClinicalTrials.gov.

Study Name	Intervention Arm	Control Arm
Investigation of the Efficiency of Pain Neuroscience Education in Patients with Chronic Pain After Breast Cancer Surgery [16]	- 4 PNE sessions - Standard physiotherapy with soft tissue mobilization, twice a week for 6 weeks - Upper extremity exercise, twice a week for 6 weeks	- 4 BME sessions - Same standard physiotherapy - Same upper extremity exercise
The Effect of Pain Neuroscience Education and Behavioural Graded Activity on Chronic Pain in Breast Cancer Survivors (BCS-PAIN) [20]	- 6 PNE sessions over 12 weeks - Behavioral graded activity	- Usual care - Informational leaflet
PI-targeted PNE + MI Compared to BIOMEDICAL Education in BCS (BCS-PI) [17]	- 1 PNE session online - 3 PNE + MI sessions over 4 weeks (45 min, one-on-one) - Leaflet PI	- 1 biomedically focused education online session - 3 biomedically focused education sessions (45 min, one-on-one)
Pain Neuroscience Education and Graded Exposure to Movement in Breast Cancer Survivors [19]	- 1 month of PNE - 2 months of gradual exposure to movement (small groups) - Informative booklet	- Usual care - Informational booklet
Pain Neuroscience Education and Therapeutic Exercise as a Treatment for Breast Cancer Survivors Living with Sequelae [39]	- 9 weeks, comprising - 3 PNE sessions - 24 therapeutic exercise sessions (16 live, 8 online)	- None
